# Pharmacologic Inhibition of CXCL10 in Combination with Anti-malarial Therapy Eliminates Mortality Associated with Murine Model of Cerebral Malaria

**DOI:** 10.1371/journal.pone.0060898

**Published:** 2013-04-05

**Authors:** Nana O. Wilson, Wesley Solomon, Leonard Anderson, John Patrickson, Sidney Pitts, Vincent Bond, Mingli Liu, Jonathan K. Stiles

**Affiliations:** 1 Department of Microbiology, Biochemistry, and Immunology, Morehouse School of Medicine, Atlanta, Georgia, United States of America; 2 Cardiovascular Research Institute, Morehouse School of Medicine, Atlanta, Georgia, United States of America; 3 Department of Pathology, Morehouse School of Medicine, Atlanta, Georgia, United States of America; University of Barcelona, Spain

## Abstract

Despite appropriate anti-malarial treatment, cerebral malaria (CM)-associated mortalities remain as high as 30%. Thus, adjunctive therapies are urgently needed to prevent or reduce such mortalities. Overproduction of CXCL10 in a subset of CM patients has been shown to be tightly associated with fatal human CM. Mice with deleted CXCL10 gene are partially protected against experimental cerebral malaria (ECM) mortality indicating the importance of CXCL10 in the pathogenesis of CM. However, the direct effect of increased CXCL10 production on brain cells is unknown. We assessed apoptotic effects of CXCL10 on human brain microvascular endothelial cells (HBVECs) and neuroglia cells in vitro. We tested the hypothesis that reducing overexpression of CXCL10 with a synthetic drug during CM pathogenesis will increase survival and reduce mortality. We utilized atorvastatin, a widely used synthetic blood cholesterol-lowering drug that specifically targets and reduces plasma CXCL10 levels in humans, to determine the effects of atorvastatin and artemether combination therapy on murine ECM outcome. We assessed effects of atorvastatin treatment on immune determinants of severity, survival, and parasitemia in ECM mice receiving a combination therapy from onset of ECM (day 6 through 9 post-infection) and compared results with controls. The results indicate that CXCL10 induces apoptosis in HBVECs and neuroglia cells in a dose-dependent manner suggesting that increased levels of CXCL10 in CM patients may play a role in vasculopathy, neuropathogenesis, and brain injury during CM pathogenesis. Treatment of ECM in mice with atorvastatin significantly reduced systemic and brain inflammation by reducing the levels of the anti-angiogenic and apoptotic factor (CXCL10) and increasing angiogenic factor (VEGF) production. Treatment with a combination of atorvastatin and artemether improved survival (100%) when compared with artemether monotherapy (70%), p<0.05. Thus, adjunctively reducing CXCL10 levels and inflammation by atorvastatin treatment during anti-malarial therapy may represent a novel approach to treating CM patients.

## Introduction

Malaria caused by *Plasmodium falciparum* infection remains a leading cause of global morbidity and mortality. An estimated 216 million cases of malaria occurred in 2010 globally, most of which were reported in sub-Saharan African children less than five years of age and resulted in 655,000 deaths [1]. *Plasmodium falciparum* in a subset of patients can lead to a diffuse encephalopathy known as cerebral malaria (CM). Cerebral malaria is a neurological complication of *falciparum* infections associated with high mortality rates and long-term morbidity. Despite appropriate anti-malarial treatment using quinine or artemisinin derivatives, CM mortalities remains high (approximately 30% in adults and 18% in children) while 25% of survivors experience long-term neurological and cognitive impairment [2]–[6]. These observations suggest that strategies targeting the elimination of parasites during CM pathogenesis may be insufficient to prevent post treatment neurological complication and death [7].

*Plasmodium berghei* ANKA infection in mice is utilized as an experimental model of CM (ECM) that exhibits several of the neurological manifestations observed in human CM [8]–[13]. Similar to human CM, *P. berghei* ANKA-infected mice develop neurological signs including hemi- and paraplegia, ataxia, and convulsions [14]–[19]. Inflammatory cytokines are up-regulated in both human CM and murine ECM brains. In addition, reduced blood flow and increased lactate production are found in the brains of both humans and mice [9], [20]. *Plasmodium falciparum* and *P. berghei* ANKA induce endothelial activation with expression of adhesion molecules such as ICAM-1 and E-selectin which are up-regulated in both human CM and murine ECM [12]. Additionally, human CM and murine ECM are characterized by severe platelet activation and accumulation within the cerebral microvasculature, coagulopathy, vascular leakage, edema, and micro-hemorrhages [21].

However, *P. berghei* ANKA-infected red blood cells may accumulate in the murine brain under certain circumstances, but whether infected red blood cells arrest is required for murine ECM development and whether the underlying mechanism qualifies as true sequestration has been a matter of debate [9]–[13]. Studies have shown little or no accumulation of infected red blood cells in the murine brain and failed to demonstrate a correlation between sequestration of infected red blood cells and ECM [13], [15], [22]. Due to this discrepancy, the value of *the P. berghei* ANKA model for screening therapeutics against human CM has been questioned [13]. Nevertheless, *P. berghei* ANKA-infected mice do exhibit substantial platelet sequestration and leukocyte marginalization [21] processes that can contribute to severe malaria [23]. While differences between murine ECM and the human CM are often emphasized [8], [24], there are also important similarities [9], [11], [12], [25], [26]. Thus, this model is suitable for studying certain aspects of the pathogenesis of human CM [12], [23], [27]–[31]. Nevertheless, we acknowledge that caution is required when translating experimental findings to clinical pathology.

Clinical studies in humans and murine models of ECM have shown that dysregulated inflammatory responses to infection and adverse effects on vascular endothelium play a central role in CM progression and outcome [20], [32]. Furthermore, accumulating evidence indicate that host immune responses modulate pathogenesis and adverse clinical outcomes of CM. These observations have led to several clinical trials that utilize adjunctive therapies to modulate host immune responses as a means of managing human CM. Unfortunately, none of these adjunctive therapies investigated to date in randomized control trials have proven to be efficacious [33]–[35]. In addition, most adjunctive therapies tried so far in murine models have been administered as prophylactics to prevent development of neurological signs. The prophylactic strategy may not be clinically relevant since most CM patients develop neurological symptoms or present at late stages of the disease at clinics. The failure of adjunctive therapies in randomized control trials suggest an urgent need for development of novel adjunctive immunomodulatory therapeutics that target and ameliorate late stages of CM in order to improve clinical outcomes and prevent or reduce neurological complication in patients who survive treatment.

Our recent studies in West Africa and South Asia demonstrated for the first time that increased plasma and cerebrospinal fluid levels of interferon gamma induced protein 10 (CXCL10) secreted by several cell types in response to IFN-γ predict fatal human CM [36]–[38]. CXCL10 is a chemokine that performs several roles, including chemo-attraction for monocytes and T cells, promotion of T cell adhesion to endothelial cells, antitumor activity, inhibition of bone marrow colony formation and angiogenesis [39]. There is a growing appreciation of the role of CXCL10 in both infectious and non-infectious causes of central nervous system (CNS) neuronal injury, dementia, and inhibition of angiogenesis [40]–[43]. Following the first report of CXCL10 association with fatal human CM, Campanella et al, [44] observed that CXCL10 was the most highly induced chemokine during murine ECM and that mice deficient in CXCL10 gene were partially protected against CM when infected with *P. berghei* ANKA [44]. Furthermore, the study revealed that CXCL10 was induced in the brain during the onset of the disease and was highly expressed in the brain of mice infected with *P. berghei* ANKA at the late stage of ECM [44]. However, the effects of CXCL10 on the microvascular endothelial or brain cells are unknown. Both human and murine studies indicate that CXCL10 is linked to the pathogenesis of CM and justifies further evaluation of its effects on brain cells since the disease is associated with the brain. Determining the effect of CXCL10 on brain cells and developing a therapeutic strategy to target its production during human CM could be a novel approach to ameliorating or reversing CM pathogenesis to improve clinical outcomes while preventing neurological complication in survivors.

Atorvastatin (ATV), a Food and Drug Administration (FDA) approved drug that inhibits 3-hydroxy-3-methylglutaryl-CoA (HMG-CoA) reductase significantly inhibits the expression of CXCL10 in humans [45]. However, the mechanism ATV utilizes to inhibit CXCL10 expression is unknown. Atorvastatin primarily inhibits HMG-CoA reductase to decrease cholesterol synthesis as well as increase the synthesis of low-density lipoprotein (LDL) receptors that result in an increased clearance of cholesterol and LDL from the bloodstream [46]. Furthermore, ATV has other beneficial effects of preventing thrombosis, protecting the vascular endothelium, stabilizing atheromatous plaque, neutralizing reactive oxygen radicals and exerting an anti-inflammatory effect [47]. In this study, we determined the effect of CXCL10 on cellular components of blood-brain barrier (human brain vascular endothelial cells [HBVECs] and neuroglia cells) by measuring apoptotic activities in vitro. Based on ATV's potent inhibitory effect on CXCL10 expression [45] and the fact that ATV is an FDA approved drug, we evaluated ATV as adjunctive therapy by repurposing it to target CXCL10 as a strategy to ameliorate CM in murine ECM. We demonstrate that CXCL10 induces apoptosis in HBVECs and neuroglia cells. We also show that ATV (25 mg/kg) significantly reduces serum CXCL10 concentration and brain inflammation and improves survival in mice with ECM. Therefore, blocking the production of CXCL10 by ATV-like agents may represent a candidate approach to the treatment and management of human CM in the future.

## Results

### CXCL10 induces caspase-mediated apoptosis in HBVECs and neuroglia cells

**Figure 1:**
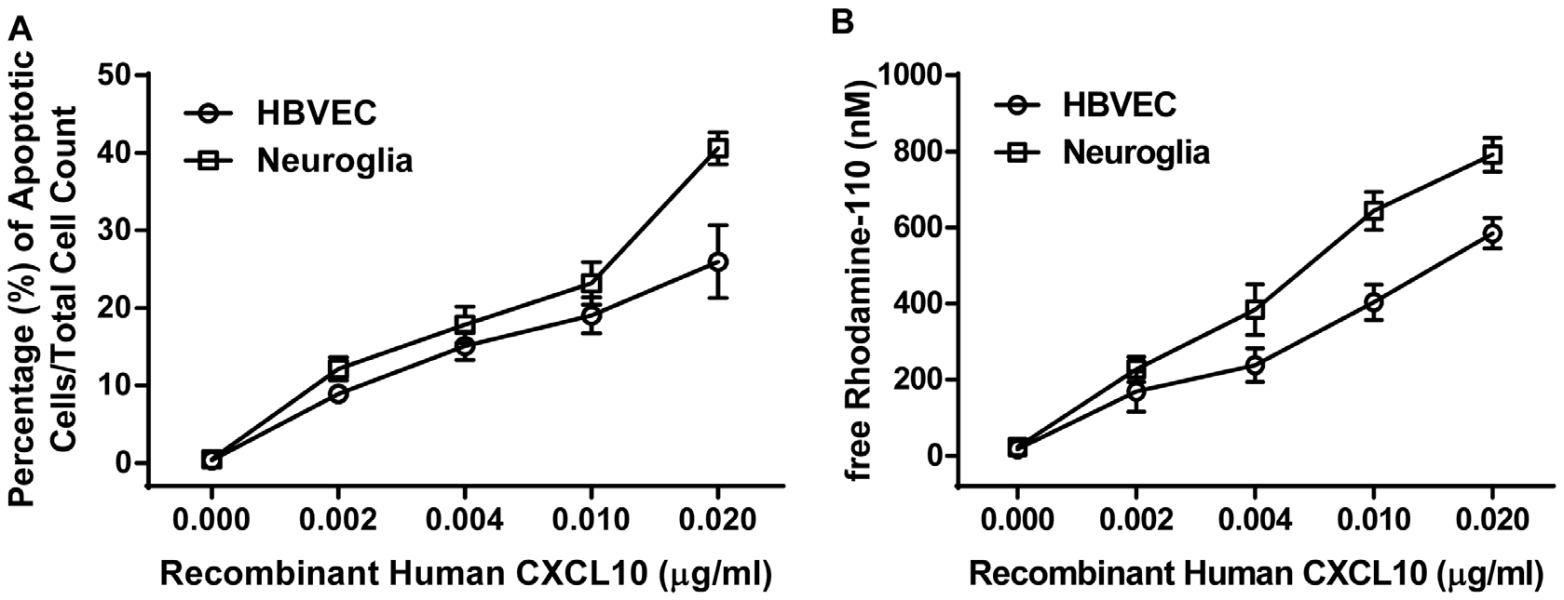
Dose-response apoptotic effect of recombinant human CXCL10 on HBVECs and neuroglia cells. (A) Shown are results of TUNEL assay of percentage of apoptotic HBVECs and neuroglia cells incubated with different concentrations of recombinant human CXCL10 for 24 hour at 37°C. (B) Homogeneous caspase assay showing HBVECs and Neuroglia cells exposed to different concentrations of recombinant human CXCL10 for 24 hours at 37°C. Subsequently, the cells were directly incubated with substrate solution for 2.5 hours at 37°C. The Relative Fluorescence Units (RFU) signal is converted to nM free Rhodamine via standard curve. The increase of the caspase activity was calculated as difference of the RFU signal of the induced cells to the RFU signal of non-induced cells. Bars represent standard deviations of three experiments.

To determine the effect of CXCL10 on brain cells, we examined the apoptotic effect of CXCL10 on HBVECs and neuroglia cells after 24 hours of exposure with different concentrations of recombinant human CXCL10 protein (rhCXCL10) by in situ terminal deoxynucleotidyl transferase dUTP nick end labeling (TUNEL) (Figure 1A) and Homogeneous Caspases Assay (Figure 1B). Recombinant human CXCL10 protein caused dose-dependent apoptosis in HBVECs (Pearson's *r* = 0.914, p = 0.029) and neuroglia cells (Pearson's *r* = 0.965, p = 0.008) which was evident 24 hours after exposure (Figure 1A). The percentage of apoptotic cells increased from 8.9% to 26% in HBVECs after treatment with 0.002 µg/ml and 0.020 µg/ml respectively of rhCXCL10. Apoptosis in neuroglia cells increased from 12% to 40.6% with CXCL10 treatment of 0.002 µg/ml and 0.020 µg/ml respectively. Figure 1B shows rhCXCL10 induced caspase activation in HBVECs (Pearson's *r* = 0.970, p = 0.006) and neuroglia cells (Pearson's *r* = 0.935, p = 0.019) treated with different concentrations of rhCXCL10. The assay detects caspases 3 and 7 and indicates a role for these caspases in CXCL10–mediated cell death. Apoptosis in both cell lines were accompanied by a significant increase in the activation of caspases.

### Combination of Artemether with Atorvastatin increases survival of mice with ECM

**Figure 2:**
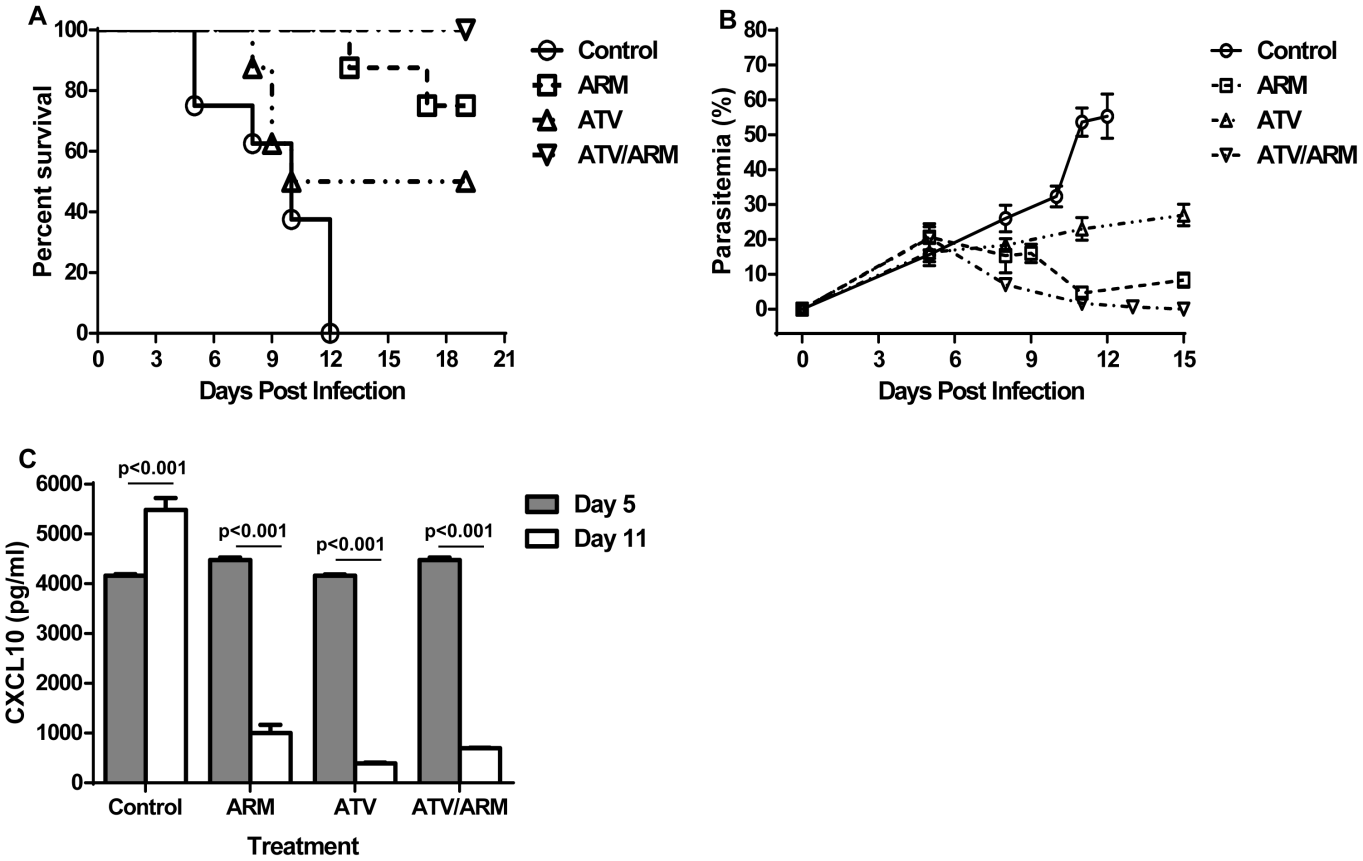
Modulating CXCL10 levels improves survival in mice with late stage ECM. (A) Survival curves: The Kaplan-Meier curves are shown for efficacy of ARM at 25 mg/kg/day (*n* = 11), ATV at 25 mg/kg/day (*n*  = 11) and ATV/ARM at 25 mg/kg/day (*n* = 11) in rescuing mice with late stage ECM. All treatments started no day 6 and ends on day 9 post infection. ARM-treated and ATV-treated mice showed survival rates of 75% and 50% respectively compared to controls (p<0.05). The survival rates improved with ATV/ARM-treated mice (100%) which are significantly superior to ARM-treated, ATV-treated or control mice (p<0.001). Survival was assessed twice daily. Significant differences in survival were assessed by Log rank test. (B) Parasitemia of *P. berghei* ANKA-infected mice after treatment. Parasitemia was monitored by Giemsa-stained blood smears using light microscopy at ×100 magnifications with an oil immersion lens. Parasitemia was checked and quantified by counting the number of parasitized red blood cells in at least 1,000 red blood cells. The experiment is a representative of three independent infections. (C) Serum CXCL10 levels of *P. berghei* ANKA-infected mice after treatment. Serum CXCL10 was measured on samples collected on day 5 and day 11 post infection (*n* = 5 per group). The results in panels B and C are mean ± the standard deviation. Mean values were determined to be significantly different using the student *t*-test. A p value of <0.05 was considered significant. ARM = artemether; ATV = atorvastatin.

The control group developed murine ECM between days 5 and 12 post-infection with mortality between 30%–100% (Figure 2A). Artemether (ARM) has been successfully used to treat *P. berghei* ANKA-infected mice [48]. Using ARM treatment alone (25 mg/kg/day from day 6 to day 9 post-infection), clinical recovery began after initiating the treatment with significant reduction in parasitemia (Figure 2B). Mortality following ARM treatment was between 15% and 25% when compared to controls (p<0.0001, Kaplan-Meier, log rank) (Figure 2A). Mice treated with ATV alone (25 mg/kg/day from day 6 to day 9 post-infection) showed reduced mortality at 50% when compared to the controls although parasitemia remained high (Figure 2A and B) suggesting that increased survival was not attributed to anti-parasitic effects but more likely modulation of host immune system.

Based on our hypothesis that the mortalities associated with late stage treatment of murine ECM was linked to the dysfunction and weakened stability of the blood-brain barrier, we have continued to search for adjunctive treatments that will stabilize the BBB at late stages of murine ECM while eliminating parasitemia and increasing survival to 100%. In this study, we have used ATV as an example of adjunctive treatment used in combination with ARM. To approximate the clinical settings, where treatment occurs after admission, mice were treated with a combination of ATV (25 mg/kg/day) and ARM (25 mg/kg/day) from day 6 to day 9 post-infection. Protection from late-stage murine ECM was significantly increased to 100% by adjunctive treatment with ATV together with ARM when compared to controls (p<0.0001), ARM alone (p<0.05), and ATV alone (p = 0.025) groups (Figure 2A).

In order to assess effects of ATV on parasitemia, we assessed parasitemia before and after treatment. There were significant differences among treatment groups when parasitemia levels were compared with saline-treated mice on day 11 post-infection (p<0.05). Levels of parasitemia for saline-treated mice increase rapidly from day 5 to day 11 post-infection by which time all the control mice had been euthanized (Figure 2A and B). Although there was steady increase in parasitemia in the ATV treatment group, the parasitemia was significantly lower (p<0.05) when compared to the control group (Figure 2B) suggesting direct ATV effects on parasitemia. Artemether treatment efficacy was demonstrated by decreased parasitemia from 21% (day 5 post-infection) to<5% (day 11 post-infection) (Figure 2B). This parasitemia was increased to 8% on day 15 post-infection. The ATV/ARM treatment group showed a decrease in parasitemia from 20% on day 5 to 0% on day 15 post-infection (Figure 2B).

### Survival of ECM mice treated with Atorvastatin is associated with decreased expression of CXCL10

We have previously established that elevated levels of CXCL10 are associated with fatal CM in human [36]–[38]. Thus, we attempted to reduce mortality in mice with ECM by testing the hypothesis that modulating CXCL10 during murine ECM will improve survival. The strategy involves repurposing ATV to modulate CXCL10 levels during murine ECM. Atorvastatin alone and ATV/ARM as well as ARM alone significantly decreased serum CXCL10 levels (p<0.001) and improved survival in ECM mice when compared with controls, p<0.05 (Figure 2A and C). The control group showed significant increase in CXCL10 on day 11, as previously reported, compared to day 5 post-infection, p<0.001 (Figure 2C).

### Atorvastatin decreases leukocyte accumulation in brain vessels

**Figure 3:**
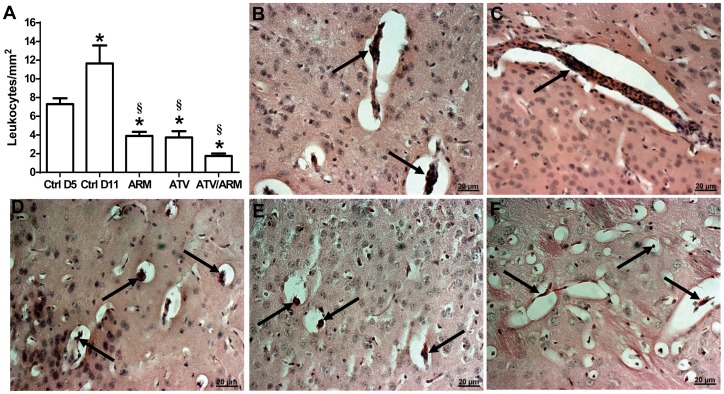
Inhibition of inflammation in the brain of P. berghei-infected mice with ECM after treatment. (A) The number of intravascular leukocytes per mm^2^ of brain area was markedly decreased after treatment. Parenchymal vessel of (B) untreated mice with ECM on day 5 and (C) saline-treated mice on day 11, plugged with leukocytes (black arrows). Parenchymal vessels of (D) ARM-treated mice, (E) ATV-treated mice and (F) ATV/ARM-treated mice showing remnant adherent leukocytes (black arrows) on day 11. The leukocytes counts are mean±standard error. A p value of <0.05 was considered significant. Asterisks (_*_) denote statistically significant change compared with ctrl D5 and section sign (§) denote statistically significant change compared with ctrl D11. Ctrl = control; D = day; ARM = Artemether; ATV = Atorvastatin.

Leukocyte adherence and accumulation in brain vessels correlate with brain inflammation and are key features of murine ECM [49]. To determine whether drug treatment was effective in reducing brain inflammation, we quantified the number of adherent leukocytes after treatment with ATV on day 11 post infection. The numbers of leukocytes per vessels and per mm^2^ decreased after treatment when compared with controls (Figure 3). Although ATV-treated mice had moderate-to-high levels of parasitemia compared to ARM-treated mice, there was a significant difference in number of leukocytes per vessels between ATV- and ARM-treated mice (Figure 3). However, ATV treatment in combination with ARM reduced the number of leukocytes adherent in the brain of the mice.

### Atorvastatin and Artemether combination treatment decreases activation of vascular endothelium and enhances blood-brain barrier integrity in murine ECM

To evaluate the effects of the treatments on endothelium activation and blood-brain barrier integrity, mRNA transcription of specific protein markers (angiopoietin-1 and 2, perforin, Fas and IFN-γ) involved in vascular endothelium activation and breakdown of BBB were assessed. Brain mRNA expression was analyzed to contrast regulated changes with those occurring at day 5 and day 11 post infection in each group.

**Figure 4:**
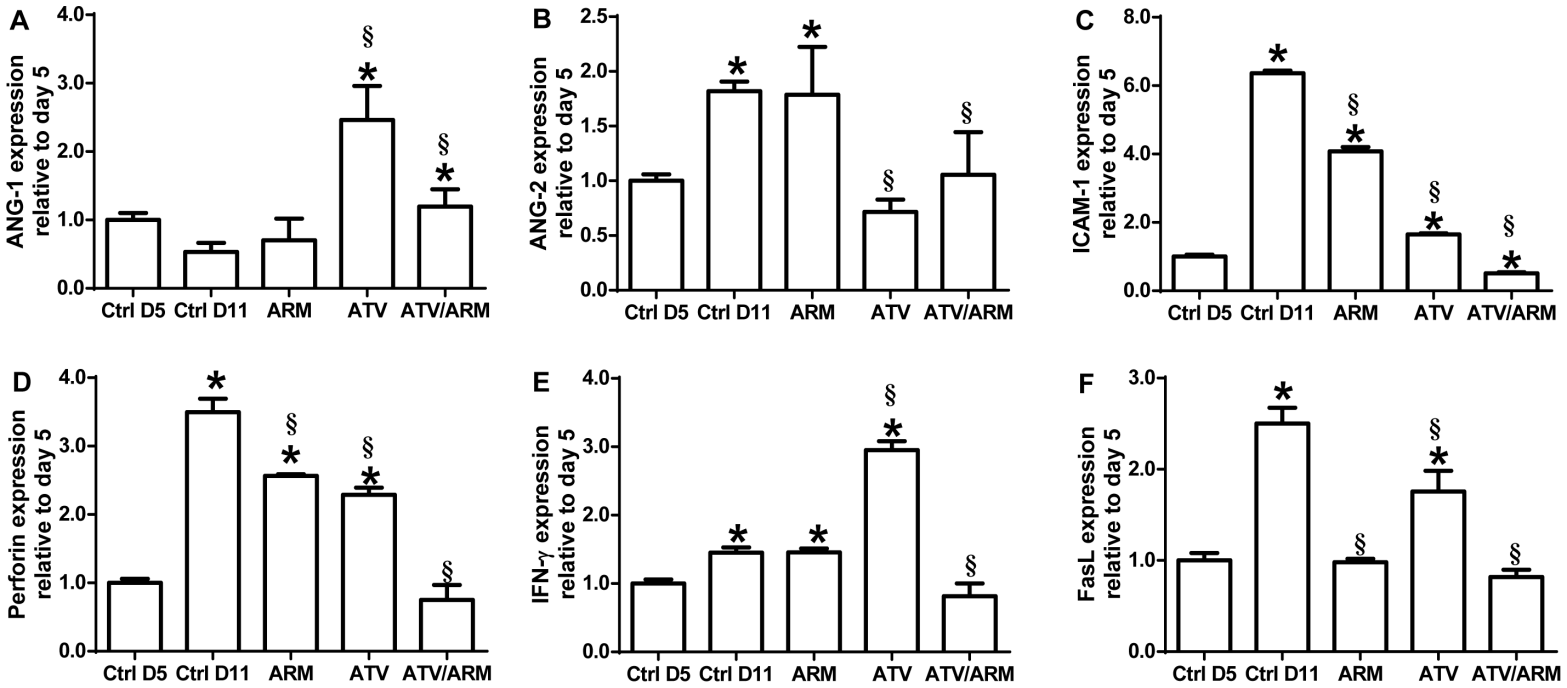
Assessment of transcriptome associated with endothelium activation and blood-brain barrier integrity. mRNA expression of angiopoietin-1 (A), angiopoietin-2 (B), ICAM-1 (C), perforin (D), IFN-γ (E), and FasL (F) in whole brain homogenates of control, ARM, ATV, and ATV/ARM-treated *P. berghei*-infected mice (*n* = 5 per group). Expression, measured by real-time PCR on day 11 post-infection, is relative to the mean expression value in control mice day 5 post-infection brain samples and normalized with HPRT values. Treatment with ATV or ATV/ARM decreases endothelium activation and improves blood-brain barrier integrity. Columns and bars represent means ± standard error. Mean expression values were determined to be significantly different using one-way ANOVA with Holm-Sidak post-tests method for all pairwise multiple comparison. A p value of <0.05 was considered significant. Asterisks (*) denote statistically significant change compared with ctrl D5 and section sign (§) denote statistically significant change compared with ctrl D11. Note the different scales used in each graph. Ctrl = control; D = day; ARM = artemether; ATV = atorvastatin.

Angiopoietin-1 and angiopoietin-2 are antagonistic factors shown to trigger endothelial cell activation [50]–[53]. Angiopoietin-1, a marker of vascular endothelial quiescence and stability, was increased in mRNA expression in brain tissue of mice treated with ATV (2.5-fold) or ATV/ARM (2-fold) at late stage murine ECM compared with saline-treated mice, p<0.001 (Figure 4A). However, there were no significant changes in angiopoietin-1 mRNA expression in saline-treated and ARM-treated mice on day 11 when compared day 5 untreated mice (Figure 4A). Angiopoietin-2, a marker that directly reflects vascular barrier breakdown was significantly reduced in expression in the brains of ATV- or ATV/ARM-treated mice when compared with saline-treated mice, p<0.001 (Figure 4B). There was a significant increase in angiopoietin-2 expression in the brain tissues of mice in saline-treated (1.9-fold) and ARM-treated (1.8-fold) mice at day 11 when compared to untreated mice at day 5, p<0.001 (Figure 4B). Conversely, ICAM-1, a marker of endothelial activation [47], showed about 6-fold induction in saline-treated and 4-fold in ARM-treated mice, p<0.001 (Figure 4C). While there was moderate increase in the level of ICAM-1 mRNA expression in the brain of ATV-treated mice (1.8-fold), there was a decrease in expression in ATV/ARM-treated mice (0.5-fold) when compared to saline-treated mice, p<0.001 (Figure 4C).

Perforin mRNA expression was significantly increased in brain tissue in saline-treated (3.5-fold), ARM-treated (2.5-fold) and ATV-treated (2.2-fold) mice compared with untreated mice on day 5 post-infection p<0.001 (Figure 4D). However, there were no significant changes in expression comparing ATV/ARM-treated mice with untreated mice on day 5 post-infection (Figure 4D). Compared to saline-treated mice there was a significant decrease in expression of perforin in the other treatment groups; p<0.001 (Figure 4D).

Both Fas and IFN-γ have been implicated in the pathogenesis of CM [54], [55]. There were significant increases in the transcription of IFN-γ mRNA in the brains of control, ARM-treated and ATV-treated mice, p<0.001 (Figure 4E). However, there were no significant changes in expression of IFN-γ mRNA among ATV/ARM-treated mice on day 11 when compared with untreated mice at day 5, (Figure 4E). Nevertheless, the expression of IFN-γ in ATV/ARM-treated mice was significantly lower when compared with saline-treated mice, p<0.001 (Figure 4E). Expression of Fas mRNA was significantly increased in saline-treated (2.5-fold) and ATV-treated (1.8-fold) mice, p<0.001 (Figure 4F). There were no significant changes in the expression of Fas mRNA in the brain tissue of ARM- and ATV/ARM-treated mice compared with untreated mice (Figure 4F). Expression of Fas mRNA in ARM- and ATV/ARM-treated mice were significantly lower when compare with saline-treated mice, p<0.001 (Figure 4F).

### Treatment with Atorvastatin modifies cytokine and chemokine expression profiles associated with murine ECM

**Figure 5:**
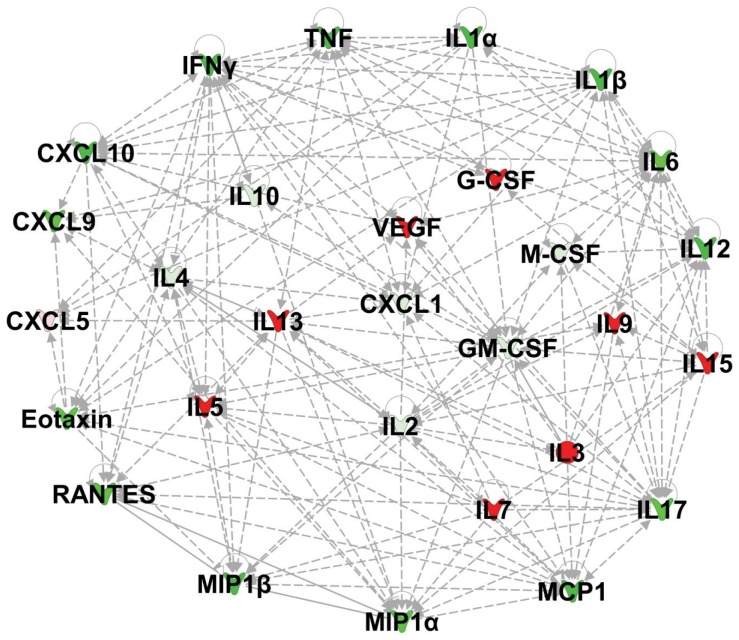
Atorvastatin regulated gene network of pro-inflammatory, anti-inflammatory and growth factors. Red-labeled genes were up-regulated and green-labeled genes were down-regulated in Atorvastatin-treated compared with saline-treated mice on day 11 of *P. berghei* ANKA infection. Color intensity signifies the degree of regulation. White labeled genes were not represented in the uploaded data set, but their connectivity was determined through network analysis.

Multiplex immunoassay of panels of cytokines and chemokines associated with murine ECM [44] were performed with milliplex MAP mouse cytokine/chemokine panel (Millipore, MA) to determine the protective effect of ATV against murine ECM and its dampening effect on CXCL10 levels. The milliplex assay compared ATV-treated and saline-treated mice on day 11 post infection. Comparing the cytokine/chemokine profiles of ATV-treated with saline-treated infected mice revealed 20 down-regulated and 9 up-regulated genes with fold changes of >1.5. Pathway analysis revealed a down-regulation of pro-inflammatory cytokines/chemokines in the panel with the exception of IL-15 and CXCL5 (Figure 5). The down-regulated cytokines/chemokines includes IFN-γ, TNF-α, IL-1α, IL-1β, IL-6, IL-12, IL-17, MCP-1, MIP-1α, MIP-1β, RANTES, eotaxin, and CXCL9 (Figure 5). Additionally, IL-2, IL-4, IL-10, GM-CSF, M-CSF, and CXCL1 were also down-regulated.

In contrast, a network of anti-inflammatory (IL-5 and IL-13) and growth factors (VEGF, G-CSF, IL-3, IL-7, and IL-9) were up-regulated in the ATV-treated compared to the saline-treated infected mice (Figure 5). Furthermore, using the Ingenuity Pathway Analysis software for core analysis it was noted that these networks of up-regulated and down-regulated cytokines and chemokines in ATV-treated mice modulates multiple biological and physiological processes during murine ECM pathogenesis. The analyses indicated that treatment with ATV enhanced or regulated biological processes such as survival and proliferation of neuroglia (p<3.27E-15), angiogenesis (p = 4.23E-20), vascular endothelial cells and blood vessel development (p<7.15E-15), and production of nitric oxide (p = 3.31E-20) (Table 1) which are essential processes required for recovery from murine ECM.

**Table 1:** The table displays biological processes or functions enhanced by Atorvastatin treatment identified by Ingenuity Pathway Analysis as significantly (p<0.05) associated with the dataset.

Biological Processes Enhanced by Atorvastatin Treatment		
Biological Process or Function	# of gene products	P-value
Survival of neuroglia	8	3.27E-15
Proliferation of neuroglia	11	5.97E-18
Angiogenesis	19	4.23E-20
Endothelial cell development	12	7.15E-15
Development of blood vessel	21	7.27E-22
Production of Nitric Oxide	14	3.31E-20

The column ’# of gene products‚ gives the number of cytokines/chemokines from the dataset obtain from the network.

### Atorvastatin regulates CXCL10 through HO-1

**Figure 6:**
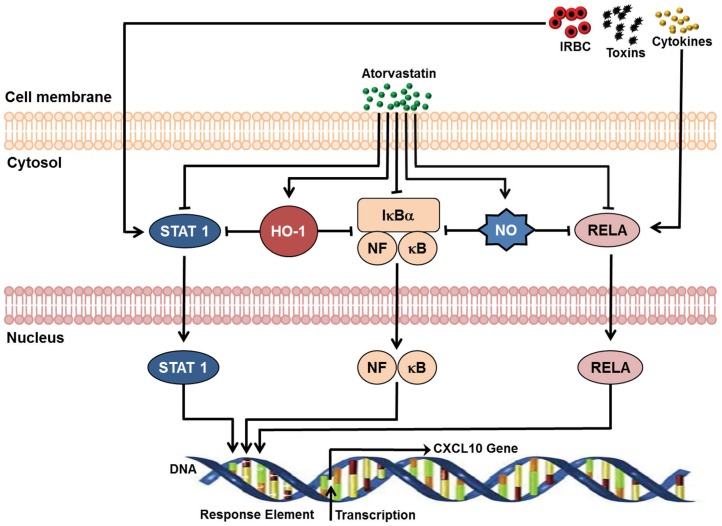
Schematic model of signaling pathways ATV utilize to modulate the expression of CXCL10 identified by Ingenuity Pathway Analysis. In response to various stimulus, transcription factors, STAT1, NFκB, and RELA, are activated resulting in the transcription of CXCL10 gene to mRNA [57]–[64]. Atorvastatin inhibit activation of RELA (NFκB3/p65) [56], [57], NFκB complex [58], [59] and STAT1 [59]. Atorvastatin increases expression of HO-1 [68] which inhibit activation of NFκB complex [69], STAT1 [70] as well as expression of CXCL10 [71]. Atorvastatin stimulates production of nitric oxide (NO) [72], which inhibit active NFκB/p65 and NFκB complex [73]–[76]. ATV = atorvastatin; IRBC = infected red blood cells; HO-1 = heme oxygenase-1; NO = nitric oxide; RELA = v-rel reticuloendotheliosis viral oncogene homolog A (avian); NFκB = nuclear factor of kappa light polypeptide gene enhancer in B-cells; STAT1 = signal transducer and activator of transcription 1.

To determine the pathway(s) through which ATV modulates the expression of CXCL10, pathway analysis was used to explore the possible relationship between ATV and CXCL10. Pathway analysis identified five possible pathways that ATV could utilize for regulating CXCL10 expression. Atorvastatin inhibits activation of NFκB3/p65 (RELA) [56], [57], NFκB complex [58], [59] and STAT1 [59] which are transcription factors that activate the transcription of CXCL10 gene to mRNA [60]–[67] (Figure 6). Atorvastatin increases expression of heme oxgenase-1 (HO-1) [68] which inhibits activation of NFκB complex [69], STAT1 [70] and expression of CXCL10 [71] (Figure 6). Atorvastatin stimulates production of nitric oxide (NO) [72], which inhibit active NFκB/p65 and NFκB complex [73]–[76]. Furthermore, nitric oxide decreases expression of CXCL10 [77] and increases induction of HO-1 [78]–[80] (Figure 6).

**Figure 7:**
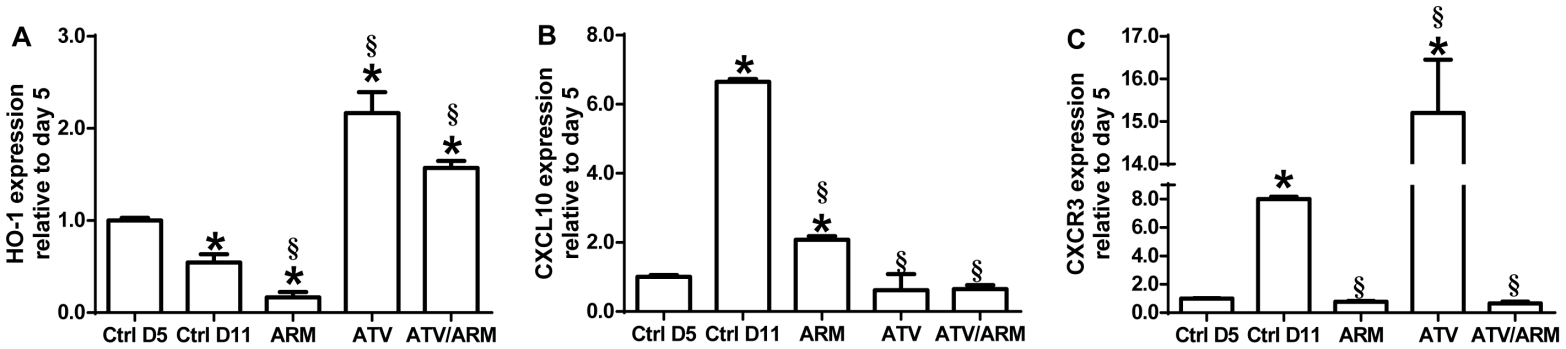
mRNA expression of HO-1 (A), CXCL10 (B), and CXCR3 (C) in whole brain homogenates of control, ARM, ATV, ATV/ARM-treated *P. berghei* ANKA-infected mice (*n* = 5 per group). Expression, measured by real-time PCR on day 11 post infection, is relative to the mean expression value in untreated mice day 5 post-infection brain samples and normalized on HPRT values. Treatment with ATV or ATV/ARM increased HO-1 expression and decreased CXCL10 expression in *P. berghei* ANKA-infection mice brain tissue. Columns and bars represent means ± standard error. Mean expression values were determined to be significantly different using one-way ANOVA with Holm-Sidak post-tests method for all pairwise multiple comparison. A p value of <0.05 was considered significant. Asterisks (*) denote statistically significant change compared with ctrl D5 and section sign (§) denote statistically significant change compared with ctrl D11. Note the different scales used on each graph. Ctrl = control; D = day; ARM = artemether; ATV = atorvastatin.

We have previously shown that heme/HO-1 and CXCL10 are directly involved in the pathogenesis of CM and that HO-1 modulates CXCL10 expression in vitro [81]. Therefore, we validated the ATV/HO-1/CXCL10 pathway by determining the expression of HO-1 and CXCL10 as well as CXCR3 in the brain tissue of the mice with late stage ECM receiving different drug treatments. Atorvastatin or ATV/ARM treatment increased HO-1 expression and decreased CXCL10 expression in the brains of the mice on day 11 when compared with untreated or saline-treated mice, p<0.001 (Figure 7A and B). Expression of HO-1 mRNA in brain tissue of the ARM group was decreased (Figure 7A). However, CXCR3, a receptor of CXCL10 mRNA expression, was increased among the ATV treatment group alone when compared with untreated or saline-treated mice, p<0.001 (Figure 7C).

## Discussion

Blood-brain barrier disruption and dysfunction are among the major features of human CM [82], [83]. Blood-brain barrier disruption is detected in children and adult patients with CM studied by in vivo imaging techniques [84]–[86], and are of prognostic value in these patients [87]. The exact cause of blood-brain barrier dysfunction and damage during human CM pathogenesis is unclear. The correlation between the number of brain petechial hemorrhages and poor clinical outcomes has led to the proposal that blood-brain barrier disruption may be partially due to vascular endothelial apoptosis caused by granzyme B and perforin-mediated cytotoxicity [88], [89]. Our previous studies indicate that CXCL10 levels are remarkably elevated in plasma and cerebrospinal fluid of patients who died from CM than those who survive the disease after treatment confirming the observation linking elevated CXCL10 in CM patients with poor prognosis [36]–[38]. Additionally, CXCL10 was an independent chemokine predictor of fatal CM in Ghanaian children and Indians who died of CM [36]–[38]. These observations in human CM were confirmed in murine studies, which revealed that mice deficient in the CXCL10 gene were partially protected against murine ECM when infected with *P. berghei* ANKA [44]. Campanella et al., [44] observed that CXCL10 was the initial chemokine induced in the brain during the onset of murine ECM and was highly expressed in the brain of mice infected with *P. berghei* ANKA at the late stage of ECM. Although, the murine malaria encephalopathy does not completely mimic human CM, both observations in human and mice demonstrate that CXCL10 is a strong indicator/predictor of CM in both human and murine ECM. However, the effects of CXCL10 on brain cells were unknown although the disease was associated with the brain tissue. Here, we have demonstrated that CXCL10 induces apoptosis in HBVECs as well as neuroglia cell in a dose-dependent manner. The apoptotic assay revealed that CXCL10-induced cell death occurs at a minimum threshold concentration of 0.002 µg/ml, which is within the physiologically relevant range of concentrations found in patients who died of CM [36], [37]. This evidence suggests that increased production of CXCL10 in CM patients may contribute to the neuro-pathogenesis and blood-brain barrier damage associated with CM. Additionally, the study indicated that neuroglia cells are more susceptible to CXCL10-induced apoptosis than HBVECs. This suggests that CM may be initiated or amplified by CXCL10 interaction with the brain cells resulting in changes in the blood-brain barrier and cerebrospinal fluid, which would allow other cytokines and malaria antigens to enter the brain compartment from which they would be normally excluded [90]. This would lead to the activation of microglia or damage to astrocytes, a phenomenon that has been observed in human CM and murine ECM [91]–[94]. Astrocytes are critical in regulating interstitial fluid milieu contributing to the maintenance of the blood-brain barrier and synthesis of neuro-protective molecules. Thus, impairment of astrocyte function may cause disruption of neuronal activity [90]. We propose that increased expression of CXCL10, a potent anti-angiogenic and apoptotic protein observed in CM patients, contributes to activation and apoptosis in endothelial cells, which perturbs the blood-brain barrier, and subsequently directly or indirectly induces neuroglia apoptosis.

Despite optimal anti-malarial treatment and advances in malaria eradication, the mortality rate associated with CM remains unacceptably high. Quinine and artemisinin derivatives (artesunate and artemether) are the drugs of choice for treatment and management of human CM [95], [96]. The rational for selecting ARM for this study is based on a previous study demonstrating that ARM was significantly effective against murine ECM than quinine, artemisinin, and artesunate [48]. However, the mortality rate was unacceptably as high as 30% despite treatment with ARM [48]. This relatively high mortality rate may be due to low efficacy of ARM against *P. berghei* ANKA or other unexplained effects of ARM on compromised blood-brain barrier at late stage ECM. Additionally, targeting parasite eradication without considering secondary effects such as tissue damage and neurological complications may be inadequate for preventing the mortalities. Thus, the need for developing innovative therapeutic strategies to effectively reduce CM-associated mortality is urgent. The current study focused on adjunctive therapy in combination with anti-malarial agents in late stage *P. berghei* ANKA murine ECM rather than early stage treatment. We used ATV adjunctively with ARM treatment of established infections and iteratively repurposed a drug selected for its ability to modulate CXCL10 and inflammatory responses in murine ECM. Using advanced bioinformatics, the study explores the effectiveness of this approach as a potential adjunctive therapy for human CM in a clinical setting.

Our previous human studies indicate that increased levels of potent anti-angiogenic and apoptotic factor CXCL10 are tightly associated with fatal human CM [36]–[38], [97], [98]. In murine ECM, removing the effect of CXCL10 by knocking out the CXCL10 gene improved survival [44], [99]. Together, these findings suggest that CXCL10 plays an important role in the pathogenesis of the disease. Applying the inhibitory role of ATV against CXCL10 in murine ECM treatment, we have demonstrated that ATV administered at the late stage of murine ECM reduced the production and effect of CXCL10 and increased survival.

Although other statins have been used in murine ECM, these studies led to significant delays in mortality or failed to improve survival [100]–[102]. Most of these statin treatments tested in murine ECM were not evaluated with or compared to effective anti-malarial drugs in the context of use in treatment of human *falciparum* malaria [101]–[104]. Rather, these statins were assessed for their anti-parasitic properties against *P. berghei* ANKA in murine ECM [101]–[104]. For example, simvastatin was evaluated for its effect on parasitemia during murine ECM with no comparison with an effective anti-malarial drug [101], [102]. These studies concluded that simvastatin had no relevant effect on parasite growth inhibition and clinical outcome of murine ECM [101], [102]. Furthermore, some studies typically examined survival benefits following administration of statin treatments to mice before the development of neurological signs. In support of this approach, a recent study using ATV alone therapeutically had no effect on survival in a murine ECM. However, a prophylactic scheme employing ATV together with mefloquine, rather than mefloquine alone, yielded a significant delay in mortality and onset of murine ECM symptoms [100]. This prophylactic strategy prevents the development of neurological signs but does not reverse established CM pathologies observed in clinical practice. Using a prophylactic may not be clinically relevant since most CM patients develop neurological symptoms or are at the late stages of the disease when they present at the clinics. The criteria used for initiating treatment in our study were strict and only mice with late stage ECM presentations that developed neurological signs were included in the study. We evaluated ATV as an adjunctive treatment along with an anti-malarial (ARM) in mice after they have developed ECM. Recently, Reis et al. [104] evaluated the therapeutic effect of lovastatin in murine ECM and determined that lovastatin decreased neuro-inflammation and prevented blood-brain barrier dysfunction in murine ECM [104]. However, lovastatin treatment failed to improve survival in mice with ECM [104]. The difference between ATV and other statins evaluated in murine ECM is that, ATV is a potent inhibitor of CXCL10 in humans [45]. In addition, our previous human studies indicated that increased levels of CXCL10 were tightly associated with fatal human CM [36]–[38], [97], [98]. Nevertheless, CXCL10 has also been found to be associated with murine ECM [44], [99]. The rational for using ATV in the current study was to assess its effect on CXCL10 expression in murine ECM. We repurposed ATV to reduce production of CXCL10 as an adjunctive therapy in murine ECM. In our study, ATV treatment given after presentation of neurological signs increased or improved survival of mice with ECM. This effect is likely due in part to reduction in anti-angiogenic and apoptotic factors including CXCL10 and enhancement of the angiogenic growth factor vascular endothelial growth factor (VEGF) which is associated with fatal human CM [36]–[38].

High levels of CXCL10 may contribute to vascular injury resulting in breakdown of the blood-brain barrier and accumulation of leukocytes to cause local hyper-inflammation [37]. Although parasite sequestration is the most common feature of patients succumbing to CM, postmortem examination has also revealed intra- and peri-vascular pathology including the presence of leukocytes within brain blood vessels in human CM [105]. These findings suggested that sequestration of host leukocytes might also contribute to the pathogenesis of some human CM cases [106]. Recent findings revealed that increased levels of several inflammatory chemokines including MIP-1α and MIP-1β and CXCL10 are associated with increased risk of severe malaria [36]–[38], [107], suggesting a role for leukocytes trafficking in etiology of human CM [106]. Similar to humans, parasitized red blood cells have been found to accumulate in brains of susceptible mice during murine ECM [108]. Since both human and murine vascular obstruction, either by parasitized red blood cells, leukocytes or by both, appears to be associated with features of CM, the *P. berghei* ANKA model is useful for analysis of the relationship between reversal of microvessel blockage and inflammation in human CM [24]. In the present study, ATV effectiveness in recovering mice with ECM was associated with a marked decrease in leukocytes accumulation in brain microvessels resulting in rapid down-regulation of brain inflammation. This suggests that ATV may play a role in reversal of vessel blockage and inflammation in human CM.

CXCL10 is pathologically involved in both human and ECM [36]–[38], [44]. CXCL10 has also been reported to play a role in CNS neuronal injury, dementia, inhibition of angiogenesis, and interfere with VEGF function [40], [41], [43]. Furthermore, CXCL10 inhibits angiogenesis and regeneration of damaged blood capillaries needed for CM recovery thereby exacerbating severe outcomes [36]. Vascular endothelial growth factor stimulates endothelial cell growth, migration, and enhances vascular permeability [37]. VEGF is important in brain tissue repair and in CNS wound healing [109], [110]. In addition, a protective effect against human CM mortality has been associated with VEGF levels as higher levels of VEGF decreased the risk of human CM mortality [37]. Together with VEGF, the angiogenic factors angiopoietin-1 (biomarker of endothelial quiescence and stability), and angiopoietin-2 (marker of vascular barrier breakdown) are major regulators of vascular inflammatory response, endothelial activation, and endothelial integrity [111]. These angiogenic factors are now proposed as informative biomarkers of disease severity and clinical response in humans [111]. Angiopoietin-1 stabilizes vascular endothelial barrier through regulation of reversible paracellular and transcellular fluid transport mechanism by opposing the action of permeability-increasing mediators such as platelet-activating factor, bradykinin, thrombin, and histamine [112], [113]. In humans, decreased angiopoietin-1 levels and increased angiopoietin-2 levels are associated with severity of CM [50]–[52], [114], [115]. Low angiopoietin-1 and high angiopoietin-2 plasma level are associated with retinopathy, discriminate human CM and severe non-cerebral malaria from uncomplicated malaria, and predict mortality from human CM [50], [52], [53], [114]. Atorvastatin treatment resulted in up-regulation of angiopoietin-1 and down-regulation of angiopoietin-2 suggesting enhanced survival provided by ATV adjunctive therapy. This indicates that administration of ATV had a significant impact on endothelial and blood-brain barrier integrity. Interestingly using bioinformatics and network analysis it was determined that ATV treatment regulates biological processes such as survival and proliferation of neuroglia, angiogenesis, and endothelia cell and blood vessels development, which are essential processes needed for recovery from human CM.

Parasite sequestration is necessary for parasite survival in the mammalian host and is important to downstream events such as obstruction of blood flow and in microvessels localized hypoxia [111]. Parasite sequestration may lead to localized release of bioactive toxins, which recruit inflammatory mediators that could contribute to onset of human CM [116]. Inflammatory cytokines contribute to disease by up-regulating the expression of adhesion molecules such as ICAM-1 to bind parasitized red blood cells to the vascular endothelium in humans [106]. ICAM-1, a marker of endothelial activation, plays a crucial role in pathogenesis of both human CM and murine ECM [117]–[119]. In humans, increased ICAM-1 levels are associated with severity of CM [117], [120]–[122]. In murine ECM, increased expression of ICAM-1 on brain endothelial cells coincide with increases in microvascular permeability while ICAM-1 deficient mice are protected from ECM [123], [124]. ICAM-1 signaling mediates endothelial activation, rearrangement of endothelial actin cytoskeleton, regulation of vascular permeability and transmigration of T cells through endothelial tight junctions into the brain parenchyma under various inflammatory conditions [118],[125]–[127]. Consistent with decreased leukocyte adherence to the micro-vascular endothelium of ATV-treated infected mice, there was a significant decrease in mRNA expression of ICAM-1 in the brain tissues of ATV-treated mice compared with saline-treated mice at day 11. Nevertheless, perforin and Fas, which are key mediators of blood-brain barrier damage [54], [55], [128], [129], were down-regulated in the brain of mice treated with ATV. Perforin and Fas are also important factors in murine ECM [54]. Perforin-mediated cytolysis occurs through apoptosis of activated endothelial cells leads to breakdown of blood-brain barrier during ECM [54], [128], [129]. Additionally, Fas-mediated apoptosis of astrocytes is a critical factor in late stage ECM pathogenesis [54], [55], [128]. Thus, both cytokines are critical to the pathogenesis of ECM.

A principal feature of human CM is the sequestration of parasitized red blood cells in the brain microvasculature and obstruction [130]–[132]. We acknowledge that murine ECM is an inflammatory syndrome with local vascular activation with inflammatory cytokines playing an essential role. There are differences and some similarities between ECM and clinical and pathological features of human CM. For instance, vascular inflammation or damage is seen in pediatric human CM cases [105]. Other studies have suggested that pro-inflammatory cytokines are involved in pathogenesis of human CM, and that increased levels of these inflammatory cytokines in the plasma and cerebrospinal fluid are tightly associated with fatal human CM [37], [38], [98], [133]. Thus, inflammation plays an essential role in fatal CM in at least a subset of clinical patients. Therefore, studies in murine ECM should provide insight into inflammatory processes and immune responses and their role in human CM pathology [24]. Proteomic analysis showed that ATV reduces production of pro-inflammatory cytokines IFN-γ, TNF-α, IL-1β, IL-6 and IL-12 and chemokine MIP-1α, MIP-1β, and RANTES. These cytokines/chemokines have all been implicated in human CM and murine ECM, but no individual molecule has been identified as a key regulator in all settings [134]. Neutralization of IFN-γ on the incidence of ECM and prevents TNF-α overproduction [135] and several studies have shown associations between TNF-α and CM [136]. However, in a clinical trial, treatment with a monoclonal anti-TNF-α antibody did not protect against human CM and exacerbated neurological sequelae [137]. Increased levels of IL-1β, IL-6, and IL-12 have also been reported in human CM [20], [138]–[141]. In the brain, MCP-1 contributes to increased permeability of blood-brain barrier and increases leukocyte infiltration into CNS during inflammation process [142]–. High levels of MCP-1 have been found in patients with neuro-inflammatory diseases, including cerebral ischemia and HIV-1 encephalitis [145], [146]. However, in the current study, ATV reduced the production of MCP-1 in the ECM model. High levels or expression of inflammatory chemokine MIP-1α, MIP-1β, and RANTES has also been associated with human CM [97], [107], [147], [148]. Interestingly, these molecules were down-regulated in murine ECM after ATV treatment.

It is widely accepted that anti-inflammatory cytokines down-regulate pro-inflammatory cytokines. Elevated levels of anti-inflammatory IL-10 have previously been reported in severe malaria [149], [150]. In addition, IL-10 has been proposed to down-regulate pro-inflammatory cytokine production, resulting in a lower incidence of cerebral symptoms in mice with ECM [151]. However, in the present study treatment with ATV in murine ECM resulted in down-regulation of IL-10 and IL-4 and up-regulation of anti-inflammatory cytokines IL-5 and IL-13.

Cerebral malaria may lead to poor neurological and cognitive outcomes in surviving patients suffering significant neurological damage leading to long-term deficits [152]. There is evidence linking CM to hippocampal damage in CM patients [153], [154]. Interestingly, ATV treatment selectively increased expression of neuro-protectants, IL-3, IL-15, and G-CSF. IL-3 exerts trophic action on hippocampal neurons and rescue hippocampal neurons from lethal ischemic damage [155]. IL-3 has been shown to be a potent inhibitor of neuronal death [156]. Pro-inflammatory cytokine IL-15 regulates neuro-immune responses by regulating T-cell and macrophage infiltration and activation after nerve injury [157], [158]. IL-15 has been shown to regulate astrocytic, microglial and neural functions [159]. G-CSF reduces apoptosis, drives neurogenesis and angiogenesis, and attenuates inflammation [160]–[163]. Moreover, G-CSF is protective in stroke, Alzheimer's disease, and spinal cord injury [164], [165]. Thus, the prevention or the reversal of neurological damage with ATV treatment may result from a combination of decreased inflammatory damage to the blood-brain barrier, increased in neuro-protectants and reduced obstructive plugging of micro-vessels. Therefore, it is likely that ATV may prevent cognitive impairment in mice recovering from ECM.

Reproduction of *Plasmodium* parasites in red blood cells causes release of free heme, which increases the expression of the anti-inflammatory enzyme HO-1 [104]. Increased expression of HO-1 prevented mortality in murine ECM and blood-brain barrier disruption, brain microvasculature congestion and neuro-inflammation [166]. Previous reports have shown that statins induce HO-1 expression [167], [168]. In the present study, we also observed that ATV increased expression of HO-1 in mice with ECM, suggesting that some of protective effects of ATV resulted from increased in HO-1 expression and potentially contributing to the decrease in the inflammatory response and oxidative damage [104]. Atorvastatin treatment resulted in up-regulation of HO-1 and down-regulation of CXCL10 in the brain of mice with ECM. In addition, HO-1 modulates CXCL10 expression [81]. Furthermore, ATV specifically modulates the expression of CXCL10 through inhibition of transcription factors (NFκB and STAT1) and increased expression of HO-1 and nitric oxide (NO) [58], [59], [68], [72], [77]. Studies indicate that NO protects against murine ECM and has been utilized in previous clinical trials as adjunctive therapy [169]–[172]. Bioinformatic analysis revealed that ATV stimulates production of NO in mice with ECM. Nitric oxide decreases expression of CXCL10 [77]. These data support the hypothesis that reducing the production of a potent anti-angiogenic and apoptotic factor CXCL10 may represent an attractive adjunctive therapy for CM and encourage further testing of atorvastatin-like molecules or drugs in the clinical setting.

The consistent failure of adjunctive immunomodulatory treatments in human CM against murine ECM has been discussed [13]. Among the many possible explanations, this may be due to interventions not targeting the range of functions central to pathophysiology of human CM. Alternatively, the ability of a drug candidate such as ATV to impact a range of functions or pathways central to pathophysiology of both human CM and murine ECM increases the likelihood that the effect will translate to human CM. For example, ATV would modulate CXCL10 expression, inflammatory mediators, and improve vascular and blood-brain barrier integrity in the brain. Therefore, while the ECM model may not completely mimic human CM, there are significant hallmarks of CM between the human and murine ECM. Further, we have identified an adjunctive therapeutic strategy that could effectively reduce CM-mortality. In the absence of an effective adjunctive therapy, we suggest the urgent need further study this drug.

Although the use of statins in children is still being debated [173], there is evidence of efficacy and safety of statins in children with familial hypercholesterolemia [174]–[176]. However, the protective effect of ATV associated with ARM treatment demonstrates that agents with ATV-like properties that target potent anti-angiogenic and apoptotic factor CXCL10 and other cytokines/chemokines involved in human CM pathogenesis are urgently needed. Thus, FDA-approved drugs for oral treatment of high cholesterol, ATV, or ATV-like molecules/drug may present a novel class of adjunctive therapeutics for CM management in intensive care units, using a combination of anti-malarial and anti-inflammatory drugs that dampen host pro-inflammatory factors. The major concern may be the cost of delivery and/or availability of atorvastatin-like agents in low-income countries where malaria is endemic. Deciphering the molecular mechanisms by which ATV prevents CM-associated mortalities, controls CXCL10 and other molecules associated with pathogenesis of the disease could provide new drug targets and efficacious ways to improve the outcome of patients suffering from CM.

## Methods

### Cell culture

Human brain vascular endothelial cells (HBVEC; Biowhittaker, Walkersville, MD) were cultured at 37°C with 5% CO_2_ in Complete Serum-free Medium Kit (Cell Systems, Kirkland, WA) supplemented with 2% fetal bovine serum (FBS; ATCC, Manassas, VA) and 100 U/ml of streptomycin, 100 U/ml of penicillin (Gibco, Grand Island, NY) and were harvested and passaged at about 70–90% confluence. At confluence, HBVECs (2×10^5^ cells/ml) were transferred into 35-mm tissue culture dish containing collagen-coated cover slip and incubated at 37°C in 5% CO_2_ for 24–48 hours.

Human neuroglia cells were obtained from the American Type Culture Collection (M059K, ATCC, Manassas, VA) and cultured at 37°C under 5% CO_2_ in a humidified atmosphere in D-MEM/F-12 medium (ATCC, Manassas, VA) supplemented with 10% heat-inactivated fetal bovine serum and 100 U/ml of streptomycin, 100 U/ml of penicillin. At about 90% confluence, the cells were transferred into a 35-mm tissue culture dish containing a collagen-coated cover slip at a density of 2×10^5^ cells/ml and incubated at 37°C in 5% CO_2_ for 24–48 hours.

### TUNEL assays

Apoptotic effect of recombinant human CXCL10 protein (R&D Systems, Minneapolis, MN) on HBVECs and neuroglia cells were determined in dose-dependent manner (0.002, 0.004, 0.010, and 0.020 µg/ml). DNA damage was determined as a means of assessing apoptosis using terminal deoxynucleotidyl transferase dUTP nick ending labeling (TUNEL) assay with In Situ Cell Death Detection Fluorescein Kit (Roche Diagnostics, Indianapolis, IN) which detect and quantify apoptosis at single cell level. Briefly, the cells were fixed in 4% paraformaldehyde in phosphate buffered saline for 1 hour and permeabilized with Triton X-100 at 4°C for 2 min and then incubated with enzyme solution and labeling solution (TUNEL reaction mixture) for 60 min at 37°C. The cells were visualized by epiflourescence on a computer-controlled Zeiss Axioskop microscope (Carl Zeiss, Thornwood, NY) linked to Magnafire 2.1C (Olympus American, Melville, NY) and Image-Pro Plus 4.1 (Media Cybernetics, Silver Springs, MD) imaging software via a charged coupled device (CCD) camera (MC 100 SPOT 60910; Photonic Science, East Sussex UK) to determine the degree of apoptosis. All experiments were done in triplicate.

### Caspase activation assay

Homogeneous Fluorimetric Caspase Assay (Roche Diagnostics, Indianapolis, IN) was used to investigate whether apoptosis occurred by a caspase-mediated mechanism in HBVECs and neuroglia cells treated with different concentration of recombinant human CXCL10 proteins (0.002, 0.004, 0.010, and 0.020 µg/ml) according to manufacturer's instructions. The release of rhodamine, which is an indicator of caspase activity in the assay, was detected by fluorescence, and fluorescence intensity was used to compare caspase activation across different concentration treatments. The homogeneous caspase assay was carried out to investigate caspase activation in HBVECs and neuroglia cells upon incubation with CXCL10 proteins for 24 hours. Briefly, the cells were incubated in caspase substrate at 37°C for 2.5 hour. The cleavage of the substrate by activated caspase and the release of fluorescence was determined fluorimetrically at 521 nm using Spectra Max 190 fluorescence microplate reader (Molecular Devices Corp., Sunnyvale, CA). The Relative Fluorescence Units (RFU) signal is converted to nM free rhodamine via standard curve.

### Ethics statement

This study was carried out in strict accordance with the recommendations in the guide for the Care and Use of Laboratory Animals of the National Institutes of Health. The protocol was approved by the Institutional Animal Care and Usage Committee (IACUC) of Morehouse School of Medicine (Permit Number 09-06).

### Mice and Plasmodium berghei ANKA parasites

Six- to eight-week-old C57BL/6J mice were purchased from Charles Rivers Laboratories (Wilmington, MA). Mice were housed at Morehouse School of Medicine in groups of no more than four per cage on 12 h light/12 h dark cycle with access to food *ad libitum* and water. Mice were allowed to adapt to their new environment for 3 days before experimentation. All experimental protocols were reviewed and approved by the Morehouse School of Medicine Institutional Animal Care and Use Committee. All procedures were performed in accordance with national regulations on animal experimentation and welfare, and the Care of Laboratory Animal Resources (CLAR) guideline was followed to minimize animal pain. The *P. berghei* ANKA was used (a kind donation of MR4, Manassas, VA; deposited by T.F. McCutchan; MR4 reagent number MRA-311). The parasite was propagated in C57BL/6J mice, and in each experiment, a fresh blood sample was obtained from a passage mouse and a suspension, containing 1×10^6^ parasitized red blood cells in 100 µl was injected intraperitoneally in each mouse of the experimental groups. Mice were checked several times daily for mortality and the development of neurological symptoms indicative of murine ECM, such as ataxia, loss of reflex, and hemiplegia. All murine ECM experiments were terminated 19 days after infection. Parasitemia were checked beginning on day 5 after infection. Parasitemia was monitored by Giemsa-stained blood smears using light microscopy at×100 magnifications with an oil immersion lens (Nikon Eclipse E200). Parasitemia was checked and quantified by counting the number of parasitized red blood cells in at least 1,000 red blood cells. After treatment, we considered dead parasites as those that presented as a mass of condensed matter inside the red blood cells and could no longer be clearly distinguish based on typical morphology.

### Clinical assessment

Simple behavioral tests (transfer arousal, locomotor activity, tail elevation, wire maneuver, contact righting reflex, and righting in arena) adapted from the SHIRPA protocol [177], [178] was used to provide a better estimate of the overall clinical status of the mice during infection. Murine experimental cerebral malaria (ECM) was defined as the presentation of one or more of the following clinical signs of neurological involvement: ataxia, limb paralysis, poor righting reflex, seizures, roll-over, and coma [48]. Clinical signs of murine ECM was evaluated and used for scoring disease severity [179]. Infected mice show presence of parasites as well as murine ECM signs on day 5 or 6 post-infection [48].

### Drug treatment regimens

The present study was designed to treat *P. berghei* ANKA-infected mice presenting clinical signs of murine ECM. This study sought to determine the efficacy of adjunctive therapy by reducing CXCL10 levels in recovery of mice with late stage ECM. The mice were treated after they were diagnosed with murine ECM on day 6 with one of the following drug regimens intraperitoneally. (i) Artemether (Sigma-Aldrich, St. Louis, MO) was prepared in coconut oil and was administered at 25 mg/kg. (ii) Atorvastatin (Sigma-Aldrich, St. Louis, MO) was dissolved in saline and delivered at 25 mg/kg.

### Experimental design

A group of 44 infected mice was used for survival experiments. To obtain significant data, 11 mice were included in each group. After diagnosis of murine ECM on day 6, mice were randomized in 4 groups. At days 6, 7, 8, and 9 post infection, 11 mice received saline (control group), 11 mice received 25 mg/kg/day of artemether (ARM group), 11 mice received 25 mg/kg/day of atorvastatin (ATV group), and 11 mice received the drug combination atorvastatin 25 mg/kg/day and artemether 25 mg/kg/day (ATV/ARM group). Each injection subsequent to the initial dose was given in the morning.

### Tissue extraction, sample preparation, and histopathology

Mice were anesthetized by isoflurane inhalation and euthanized on days 5 and 11 after infection. Mice were perfused with 10 ml of cold sterile phosphate buffered saline to clear vessels of blood and brains were collected and processed for histology. Brains were stored in formalin for fixation, embedded in paraffin, and sectioned at 10 µm. Sagittal sections of the brain were mounted on glass slides and stained with hematoxylin-eosin (H&E). The number of leukocytes in each vessel of each section was quantified by using an ocular grid calibrated with a ×400 magnification in a Zeiss Axioskop 2 plus microscope (Carl Zeiss Microscopy, NY). The whole area of each section was similarly quantified with the grid calibrated at×40 magnification. Pictures were taken with a Zeiss AxioCam HRc camera (Carl Zeiss Microscopy, NY).

### Measurement of cytokine and chemokine levels in serum

To determine the effect of drug treatments on CXCL10 as well as levels of known pro- and anti-inflammatory cytokine and growth factors associated with CM, serum cytokines were measured from pre-treatment (day 5) and post-treatment (day 11) blood draws using a commercially available multiplex bead-based cytokine assay (Millipore, MA) coupled with the Luminex system (Austin, TX). Thirty-two analytes (CCL1, G-CSF, GM-CSF, IFN-γ, IL-10, IL-12 (p40), IL-12 (p70), IL-13, IL-15, IL-17, IL-1α, IL-1β, IL-2, IL-3, IL-4, IL-5, IL-6, IL-7, IL-9, CXCL10, CXCL1, LIF, CXCL5, M-CSF, CCL2, CXCL9, CCL3, CCL4, MIP-2, CCL5, TNF-α, VEGF) were analyzed. Biological factor levels were measured using optimal concentrations of standards and antibodies according to the manufacturer's instructions. The data was analyzed using Ingenuity Pathway Analysis software.

### RNA extraction and reverse transcription (RT)-PCR

Whole brain tissue from each group of mice were collected and immediately homogenized in Trizol reagent (Life Technologies, Gaithersburg, MD). Total RNA was extracted using RNeasy Mini Kit (QIAGEN, CA). Briefly, chloroform (0.2 ml) was added to the homogenate, and the lysate mixed thoroughly. After centrifuging at 12,000×g for 20 min at 4°C, the aqueous layer was transferred to a new tube. RNA was precipitated with 500 µl of isopropanol and pelleted by centrifuging at 12,000×g for 20 min at 4°C. Any contaminating genomic DNA was removed by DNase treatment using RNase-Free DNase Set (QIAGEN, CA) according to manufacturer's instructions. DNase-treated RNA samples were subsequently stored at−80°C until ready to use.

Reverse transcription of RNA samples was performed prior to quantitative PCR. cDNA was synthesized from up to 2 µg of total RNA iScript™ cDNA Synthesis Kit (Bio-Rad Laboratories, Hercules, CA) using Multigene Gradient Thermal cycler (Labnet International, Inc.). The resulting cDNA was diluted 1:10 by addition of 180 µl of distilled water for quantitative PCR analysis. The primer sequences used for quantitative PCR are described in Table 2.

**Table 2:** Primer sequences used.

Target gene or mRNA	Primer 5′ - 3′	
	Forward	Reverse
HPRT	GCTTTCCCTGGTTAAGCAGTACA	CAAACTTGTCTGGAATTTCAAATC
ICAM-1	GCCTCCGGACTTTCGATCTT	GTCAGGGGTGTCGAGCTTTG
Perforin	TTGGCCCATTTGGTGGTAAG	AGTCTCCCCACAGATGTTCTGC
IFN-γ	CAGCAACAGCAAGGCGAAA	GCTGGATTCCGGCAACAG
FasL	ACCACCACCTGTGTCACCACTA	CACCGGTAGCCACAGATTTGT
CXCL10	GACGGTCCGCTGCAACTG	GCTTCCCTATGGCCCTCATT
CXCR3	AATGCCACCCATTGCCAGTAC	AGCAGTAGGCCATGACCAGAAG
HO-1	GCCACCAAGGAGGTACACAT	CTTCCAGGGCCGTGTAGATA
ANG-1	ATGCTGTTCAAAACCACACG	TTTCAAGTCGGGATGTTTGAT
ANG-2	ATGTGGTGCAGAACCAGACA	GCAGCTCGAGTCTTGTCGTC

The quantitative real-time PCR assay was performed using Bio-Rad C1000 thermal cycler (Bio-Rad Laboratories, Hercules, CA). Approximately 20 ng of cDNA was used in each 25 µl PCR reaction using the Bio-Rad iQ™ SYBR® Green Supermix (Bio-Rad Laboratories, Hercules, CA) and 50 µM of each primer. After a 15 min incubation at 95°C, amplification was achieved by 39 cycles of a 15 s denaturation incubation at 95°C, followed by a 30 s annealing incubation at 55°C and 30 s extension incubation at 72°C. The identity and purity of the PCR product was confirmed by using dissociation curves and by checking the melting temperature of the PCR product, independently of the PCR reaction. To determine the relative amount of target cDNA present, the cycles to threshold (Ct) values of the target genes were compared with the basal expression of the housekeeping gene, hypoxanthine guanine phosphoribosyltransferase (HPRT). The average amount of HPRT present in each mouse group was used to normalized the quantity of target mRNA sequence against total RNA in each reaction. The differences in Ct values between HPRT and target gene of day 11 after infection of each group were compared with day 5 after infection untreated control samples to determine the relative change in mRNA expression.

### Pathway analysis

To determine the regulatory pathways linked to CXCL10 and modified by ATV as well as its effects on disease processes, network and pathways analyses were performed using the Ingenuity Pathway Analysis (IPA) software (Ingenuity Systems, Inc., Redwood City, CA; www.ingenuity.com). In addition, we identified up- and down-regulated genes that are significantly associated with ATV treatment on day 11 after infection relative to saline treatment on day 11 after infection. Briefly, each gene identifier was mapped to it corresponding gene object in the Ingenuity Pathway Knowledge Base. Day 11 protein fold-change of ATV-treated mice relative to saline-treated mice was overlaid onto global molecular network in the Ingenuity Pathway Knowledge Base. These networks were ranked based on the significance score value calculated by the Ingenuity Pathway Analysis software. Networks of focus genes were algorithmically generated based on their connectivity and their association with biological function and/or disease most significant for the dataset. The Fisher's exact test was used to calculate p-values relative to the probability that each biological function and/or disease assigned to that dataset was due to chance alone. A graphical presentation of the molecular relationships between genes/gene products for each of the top ranked networks was generated where gene or gene products are represented as nodes and the biological relationship between nodes is represented by a line. The nodes are colored red to indicate up-regulation, green to indicate down-regulation and gray to indicate genes, which did not reach threshold for expression or fold change. The intensity of the color of the node corresponds to the degree of change in the gene expression.

### Statistical analysis

Results were expressed as means ± standard deviation (SD) from at least three separate experiments performed in triplicate unless otherwise stated. Differences between means among the treatment groups were analyzed by using student two-tailed *t*-test or one-way analysis of variance with Holm-Sidak post-tests method where appropriate. Correlations between recombinant human CXCL10 and apoptotic cells or free Rhodamine-110 were performed with Pearson correlation analysis. Kaplan Meier log-rank test was used to compare the different survival curves. A p-value of <0.05 was considered significant. SigmaPlot (version 11.0) with SigmaStat (version 3.5) integration (Chicago, IL) and SAS version 9.2 (Cary, NC) software for windows were used for statistical analysis. GraphPad Prism version 6 (La Jolla, CA) for windows was used to generate all the graphs.
